# Cannabinoid consumption among cancer patients receiving systemic anti-cancer treatment in the Netherlands

**DOI:** 10.1007/s00432-022-04085-z

**Published:** 2022-07-02

**Authors:** Yrina Oelen, Sven Revenberg, Judith de Vos-Geelen, Robin van Geel, Janna Schoenmaekers, Marieke van den Beuken-Everdingen, Liselot Valkenburg-van Iersel

**Affiliations:** 1 Division of Medical Oncology, Department of Internal Medicine, GROW-School for Oncology and Developmental Biology, Maastricht University Medical Center, Maastricht, The Netherlands; 2 Department of Clinical Pharmacy and Toxicology, Maastricht University Medical Center+, Maastricht, The Netherlands; 3 CARIM School for Cardiovascular Disease, Maastricht University, Maastricht, The Netherlands; 4 Centre of Expertise for Palliative Care, Maastricht University Medical Centre (MUMC+), Maastricht, The Netherlands

**Keywords:** Cannabinoids, Cancer, Systemic therapy, Clinical practice

## Abstract

**Purpose:**

Despite the inconclusiveness regarding health effects of cannabinoids among cancer patients, studies from non-European countries suggest that the medical-intended consumption of such products by this patient group is significant. The current study analyses cannabinoid usage among oncology patients receiving systemic treatment in the Netherlands.

**Methods:**

The current study included adult patients receiving intravenous systemic therapy at Maastricht Comprehensive Cancer Centre, for a solid malignancy. Participants were asked to complete an anonymous questionnaire including questions on demographic variables, clinical variables and cannabinoid consumption.

**Results:**

A total of 153 patients with solid cancer were included in this study. Almost 25% reported usage of cannabinoids for medical purposes, with 15% of the patients currently using the substance. Additionally, 18% of non-users considered future medical usage. In 48% of the cases, consumption was reported by the oncologist. The proposed anti-cancer effect was reported by 46% of the users as motivation for consumption. Current users were mainly palliative patients and 54% of the users were undergoing immunotherapy. Intention of treatment and type of therapy were predictive factors for consumption. Cannabinoid-oil was the most frequently used way of consumption.

**Conclusion:**

This study underlines the high number of cannabinoid users among oncology patients in the Netherlands in presumed absence of clinical guidance. It highlights the essence of a pro-active role of the clinician, assessing cannabinoid usage and educating the patients on the most recent evidence regarding its potential benefits and risks. Further studies on clinical decision making and efficacy of cannabinoids are recommended, to improve clinical guidance.

**Supplementary Information:**

The online version contains supplementary material available at 10.1007/s00432-022-04085-z.

## Introduction

As a consequence of the globally increased public interest in the medical use of cannabinoids, these substances gain extensive amount of attention in the media (Bridgeman and Abazia 2017). Through these media, their alleged beneficial effects for a variety of diseases are widely propagated (Shi et al. 2019). In line with the increased interest in these products, over-the-counter sale of freely accessible cannabis-derived products, such as cannabinoid oils, has increased (McGregor 2020). Additionally, statistics from the Netherlands show an increase in medical prescription of both concentrated cannabis-derived oils and herbal cannabis (de Hoop 2018). The numerous anecdotal success stories about its potential analgesic and anti-emetic effects, could make cannabinoids appealing for a variety of patient populations, among which oncology patients, who often present with pain and nausea (Blake et al. 2017; Bouquié et al. 2018; Pearce et al. 2017). The great adverse impact of cancer-related symptoms on quality of life, the potential intolerability to standard treatment or irresponsiveness to traditional analgesic therapy, could contribute to the susceptibility of cancer patients more to rely on alternative therapeutic options that are announced in the media, such as cannabinoids (Blake et al. 2017), especially when these products are freely accessible.

Even though anecdotal evidence on the analgesic properties of cannabinoid substances is abundant, high-quality clinical trials validating these effects in cancer patients, are lacking (Whiting et al. 2015). Some evidence exists suggesting that oromucosal application of cannabinoid extracts beneficially impacts pain in advanced cancer patients (Johnson et al. 2010); Noyes et al. 1975). However, these studies often present with limited statistical power (Blake et al. 2017), similar to the review of Wang et al. (2021), who reported a minimal effect of both medical cannabis extracts and cannabinoid oil on pain relief in cancer- and non-cancer patients (Wang et al. 2021). Additional evidence shows no significant change in pain relief through medical cannabis extracts among cancer patients, compared to standard pain medication, thereby questioning the relevance of introduction of cannabinoids into clinical practice for pain management (Campbell et al. 2001). The same holds for the proposed anti-emetic effects of cannabinoids. Several small studies demonstrate a superior efficacy of medical cannabis extracts over placebo on prevention and treatment of chemotherapy-induced nausea and vomiting (CINV) (Grimison et al. 2020; Kramer 2015; National Academies of Sciences Engineering and Medicine 2017), but limited RCTs are available, the power of the studies is questionable and outdated anti-emetics are used as a control (Chow et al. 2029; Mersiades et al. 2018; National Academies of Sciences Engineering and Medicine, 2017). Despite the abundance of systematic reviews on the effectiveness of cannabinoids, the strength of their conclusions are limited due to scarce high-quality underlying research (Pratt et al. 2019). Hence, evidence supporting the proposed effects of cannabinoids among cancer patients, although promising, is minute.

In addition to the lack of proof for beneficial effects of cannabinoid use among oncology patients, many uncertainties persist regarding interactions between anti-cancer agents and cannabinoids, which raises concerns about the safety of cannabinoids in patients with cancer undergoing systemic treatment (Bouquié et al. 2018). Recent observational studies show a significant association between the use of cannabinoids during immunotherapy and worse overall survival, potentially due to interference of the anti-inflammatory effect of cannabinoids with responsiveness to immune checkpoint inhibitors (Biedny et al. 2020). Furthermore, time to progression of the tumor (TTP) is suggested to be shorter. Despite the lack of prospective evidence of the effect of cannabinoids during immunotherapy, these observational data suggest that exposure to cannabinoid substances during immunotherapy should be approached carefully (Bar-Sela et al. 2020).

Notwithstanding the ambiguity concerning the effectiveness and safety of cannabinoid usage in oncology patients, data from abroad suggest usage of cannabinoid substances among this patient group to be significant (Donovan et al. 2019, 2020). Although the degree of consumption was widely spread over different studies and different countries (Martell et al. 2018; Rajasekhara et al. 2020; Waissengrin et al. 2015) prevalences up to 25% were reported (Pergam et al. 2017). This included both patients under best supportive care as well as on active treatment, the latter independently being proposed as a predictive factor for consumption (Martell et al. 2018). Reported reasons for use varied and included mainly physical cancer-related symptoms (Pergam et al. 2017), primarily cancer-related nausea and pain. Nevertheless, several studies showed that about one third of the cannabinoid users, consume cannabinoid substances for presumed curative purposes (Mousa et al. 2019; Rajasekhara et al. 2020). These are disturbing findings, since no clinical trials to date are supporting cannabinoid use for this purpose (Mousa et al. 2019). Such information on cannabinoid use among Dutch oncology patients, however, is not available and results derived from other countries cannot directly translate to the Netherlands, due to different legal status of cannabinoids (Donovan et al. 2019). Hence, not much is known about cannabinoid use among oncology patients in the Netherlands, nor about characteristics of its users, patterns of consumption, reasons for consumption, and potential perceived effects.

The rapid growth in public interest in cannabinoid usage, its increasing social acceptance and private accessibility, the significant use of cannabinoids for medical purposes among oncology patients in other countries, and yet simultaneous inconclusiveness of the available knowledge regarding its health effects (Whitcomb et al. 2019), indicate a need to understand the utility of cannabinoids among cancer patients undergoing systemic treatment in the Netherlands (Pergam et al. 2017). This is especially important in light of the lack of proven clinical evidence for its efficacy and concurrent potential curative believes among patients, while potential risks concerning interference with particularly immunotherapy, cannot be excluded. Gaining insight in the cannabinoid use among oncology patients undergoing systemic treatment in the Netherlands, will increase awareness among doctors and help identifying those patients who are most likely to use cannabinoids. This supports doctors in managing patient expectations for use, through increasing the understanding of potential risks, benefits and uncertainties (Donovan et al. 2019, 2020).

Therefore, this research is aimed at gaining insight in cannabinoid usage among oncology patients receiving systemic therapy in the Netherlands. It aims to identify the demographic and clinical characteristics of patients, associated with cannabinoid consumption, in addition to determinants of use and perceived effects. Thereby, this study makes a novel contribution to the existing literature on cannabinoid usage among cancer patients.

## Methods

### Setting

This study is conducted at the Maastricht University Medical Centre (MUMC +). This study was exempted from the Human Subjects Act by the medical-ethical evaluation board of the academic hospital Maastricht and Maastricht University. Data was collected over a 10-week time period, spanning from December 2020 until February 2021.

### Participants

The study aimed at including 150 patients. Patients were eligible for the study, if they were at least 18 years of age and received intravenous systemic treatment at the outpatient facility of Maastricht University Medical Centre (MUMC +) for any type of solid cancer. Patients treated with curative as well as patients treated with palliative intent were applicable for inclusion in the study. Intravenous systemic therapy could be the solitary treatment for the malignancy as well as being administered as a (neo) adjuvant treatment. Exclusion criteria were the incapability of speaking Dutch and not being capable to independently answer questions from the survey.

### Study procedure

All patients fulfilling study criteria were approached at our outpatient facility. After written informed consent, data were collected by means of a survey. Recruitment of patients occurred parallel to data collection.

### Data collection

The survey contained questions concerning clinical features and sociodemographic variables (sex, age, education, ethnicity, smoking history, comorbidities, current medication, type of cancer, anti-cancer treatment and intention of treatment) and details on cannabinoid usage (current or past usage, the intention of usage, characteristics of usage). Based on this, patients were allocated to either of the 5 categories, (1) never used cannabinoids, (2) recreative use of cannabinoids in the past, (3) medical use of cannabinoids in the past (4) current recreative use of cannabinoids, and (5) current medical use of cannabinoids.

Patients of category 1 and 2, were asked for the likelihood of future medical usage of cannabinoids, motivation for future medical usage and characteristics of potential future use in terms of the supposed product, dosage and frequency of consumption. Patients of category 3, were asked for reasons for both starting and stopping the use of cannabinoids for medical purposes and their perceived effects, in addition to characteristics of consumption. Patients currently using cannabinoids for recreative purposes (group 4) were asked about current frequency and duration of usage, and potential effects on symptoms related to their cancer. Patients currently using cannabinoids for medical purposes (group 5), were asked about the consumed product, current frequency and duration of usage, motivation of usage, potential curative believes, and their perceived effects.

### Data analysis

The rate of cannabinoid use was determined for the different usage groups. Per group, demographic variables, utility characteristics, potential motivational aspects and potential perceived effects were analyzed through descriptive statistics, resulting in percentages and absolute values. Multivariate logistic regression on age, educational level, smoking history, type of cancer, intention of treatment and type of treatment, was applied to identify predictive factors for consumption. A *p*-value of < 0.05 indicated significance.

## Results

A total of 153 patients signed informed consent. One person withdrew informed consent for non-specified reasons. Therefore, 152 patients are included in the analysis.

### Demographics of the participant

The mean age of the participants was 63.3 ± 10.4 years (SD). Men and women were evenly represented in the study population (*n* = 80; *n* = 72). The vast majority of the participants had a Dutch ethnicity (*n* = 133, 91.1%). About 38% of the participants reported having at least a college degree (*n *= 57) (Table 1).

**Table 1:** Demographic variables of participants. Presented for total population and users of cannabinoid substances, separated for current and previous users

	Total population (*n* = 152)	Total users (*n* = 35)	Current users (*n* = 23)	Previous users (*n* = 15)
Age (mean), years	63.3 ± 10.4	61.2 ± 9.3	63.3 ± 9.4	57.2 ± 7.46
Sex				
Male	80 (52.6%)	17 (48.6%)	13 (56.6%)	5 (33.3%)
Female	72 (47.4%)	18 (51.4%)	10 (43.5%)	10 (66.7%)
Education				
College degree	57 (37.5%)	11 (31.4%)	8 (34.8%)	4 (26.7%)
Type of cancer				
Breast	16 (10.5%)	4 (11.4%)	1 (4.3%)	4 (26.7%)
Gastrointestinal	25 (16.4%)	4 (11.4%)	2 (8.7%)	2 (13.3%)
Urological	12 (7.9%)	5 (14.3%)	4 (17.4%)	1 (6.7%)
Lung	58 (38.2%)	15 (42.9%)	11 (47.8%)	6 (40.0%)
Melanoma	10 (6.6%)	1 (2.9%)	1 (4.3%)	0 (0.0%)
Gynecological	12 (7.9%)	1 (2.9%)	1 (4.3%)	0 (0.0%)
Head and neck	17 (11.2%)	4 (11.4%)	2 (8.7%)	2 (13.3%)
Other	2 (1.3%)	1 (2.9%)	1 (4.3%)	0 (0.0%)
Treatment				
Chemotherapy	63 (41.4%)	13 (37.1%)	8 (34.8%)	7 (46.7%)
Immunotherapy	53 (34.9%)	19 (54.3%)	14 (60.9%)	6 (40.0%)
Targeted therapy	7 (4.6%)	2 (5.7%)	0 (0.0%)	2 (13.3%)
Combined chemotherapy/ immunotherapy	17 (11.1%)	0 (0.0%)	0 (0.0%)	0 (0.0%)
Combined chemotherapy/targeted therapy	10 (6.6%)	1 (2.9%)	1 (4.3%)	0 (0.0%)
Combined immunotherapy/targeted therapy	2 (1.3%)	0 (0.0%)	0 (0.0%)	0 (0.0%)
Intention of treatment				
Palliative	99 (65.1%)	28 (80/0%)	19 (82.6%)	11 (73.3%)
Curative	53 (34.9%)	7 (20.0%)	4 (17.3%)	4 (26.7%)
Smoking history				
Current	13 (9.0%)	7 (21.9%)	4 (20.0%)	4 (26.7%)
Previous	94 (64.8%)	22 (68.8%)	14 (70.0%)	10 (66.7%)
Never	38 (26.2%)	3 (9.4%)	2 (10.0%)	1 (6.7%)

Out of the 152 participants, 65.0% was treated with palliative intent. Over 40% (*n* = 65) of the participants received immunotherapy. Different types of cancers were included. Lung tumors were the most prevalent (Table 1).

### Prevalence of cannabinoid usage

In the current study population, 15% (*n* = 23) of the participants reported current use of any type of cannabinoid for medical purposes. Three of the current users, also reported previous consumption of cannabinoids, unrelated to the current episode of consumption. In 48% of the patients using cannabinoids for medical purposes, it was reported by the clinician in the patient files. In total, 23.0% of the participants were known with the use of cannabinoids for medical purposes, either currently or in the past. Additionally, 15.8% of the participants (*n* = 24) reported previous use of cannabinoid substances for recreational purposes, while 3 participants reported current recreative use of cannabinoids (2.0%). Among the participants who never used cannabinoids for medical purposes in the past, nor currently using it with this intent, 22.5% considered future usage for medical reasons.

### Characteristics of users

Cannabinoid consumption was equally divided over gender (male *n* = 17, female *n* = 18). The mean age of the users was 61.2 ± 9.3 years, which is comparable to the age of the complete study population.

Of the current or previous users of cannabinoids for medical purposes, 80% were treated with palliative intent (*n* = 28). Multivariate analysis showed intention of treatment to be a predictive factor for consumption (*p* = 0.02, OR = 0.334).

Out of the current or former users of cannabinoids for medical purposes, 19 participants received treatment containing immunotherapy (54%), whereas 40% of the participants received treatment containing chemotherapy (*n* = 14). Only 2 patients received solely targeted therapy (6%) (Table 1). Type of treatment was shown to be a predictive factor for consumption (*p* = 0.026, OR = 1.564).

Current or former users reported a smoking history of 32.5 ± 27.3 packyears compared to 23.8 ± 27.1 packyears in the general study population (Table 1, *p* = 0.3).

### Features of utility

**Fig. 1:**
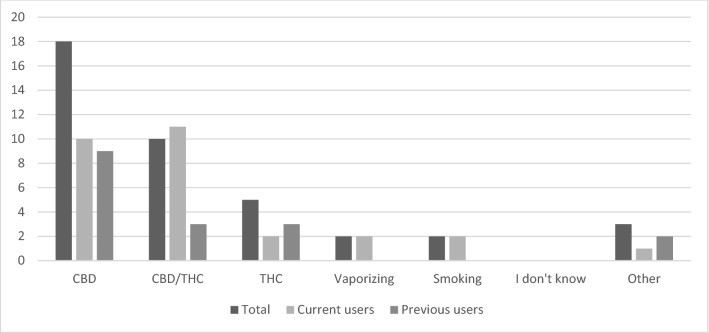
Methods of consumption of cannabinoids with medical intentions, for total group and separated for current and past users

CBD-oil was used by 18 patients (51.4%), whereas combined CBD/THC oil was used by 10 patients (29%). Only 2 patients reported smoking as the most favorable way of consuming cannabinoids (Fig. 1).

Among current users, consumption of CBD-oil and combined CBD/THC oil, was equally divided (*n* = 10, *n* = 11). Of the current users, 78% were consuming cannabinoids daily. Most of the users (52%) reported a consumption frequency of once a day, while 26% of the current users reported a consumption frequency of multiple times a day. The former users reported mainly consumption of CBD-oil (*n* = 9, 60.0%). Only 2 patients reported trying more than 1 type of cannabinoid, whereas the majority of the patients who previously used cannabinoids with medical intent, tried only one type of consumption (Fig. 1).

Most of the patients who are currently using cannabinoids retrieve the substance from friends or family (*n* = 7, 30.4%). Only one of the participants reported to get the cannabinoid substances prescribed by the doctor. Of the participants who have not been in contact with cannabinoids for medical purposes, and who considered starting it with these reasons, 48% (*n* = 13) reported their most favorable source of cannabinoids to be the prescription by the doctor, whereas uncontrolled sources were less preferred.

### Motivation for consumption

**Fig. 2:**
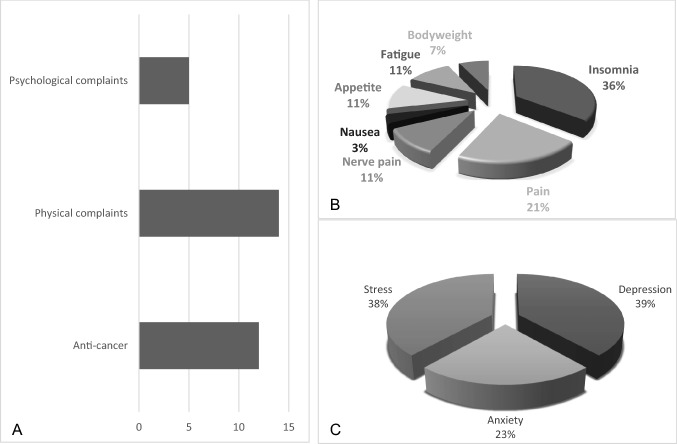
Reported reasons for cannabinoid use among current users with medical purposes. Reasons for use were not mutually exclusive. **a** Motivation separated for psychological, physical or anti-cancer believes. **b** Reported symptoms for usage, categorized for physical complaints. **c** Reported reasons for usage, categorized for psychological complaints

Almost half of all users, including the current and previous users, reported the intent to treat or cure cancer, as a motivation for their consumption (*n* = 16, 45.7%). Among current users, this percentage was 52.2% (Fig. 2a), whereas among previous users, this percentage was 26.7% (Appendix 1, Fig. 3). The majority of the patients started the use of cannabinoid substances after their diagnosis.

Regarding physical symptoms, the most common symptom for consumption in former users was pain (Appendix 1, Fig. 3). Two of these patients reported a pain rating score equal or higher than 5 on the Numeric Pain Rating Scale (NRS) during time of assessment, equal to current users. Among current users, sleeping problems were the most reported symptom as reason for cannabinoid consumption (Fig. 2b; Appendix 1, Fig. 4). Psychological complaints were more often reported as a motivation among current users, compared to past users (Appendix 1, Figs. 3, 4).

Of the non-users who considered potential future usage for medical reasons (*n* = 27), 74.1% reported considering using cannabinoids for pain, while 25.9% of them considered future usage for anti-cancer purposes. Only four patients (15%) reported potential future usage of cannabinoids for psychological purposes.

### Perceived effects

The general effect score, rated from 1 (no effect) till 4 (great effect), averaged over all physical and psychological symptoms, was 3.1. The participants who used cannabinoids for medical purposes in the past, reported an average effect score of 1.6. In line with this, over 47% (*n* = 7) of the previous users reported lack of effect as the reason to stop using cannabinoids. Other reasons for stopping the consumption of cannabinoids were, among others, side effects (*n* = 3), costs of the cannabinoids (*n* = 1) and advise of the doctor (*n* = 1). On the contrary, only one of the recreative users reported effectiveness of smoking cannabis on nausea and stress levels.

## Discussion

This research aimed at gaining knowledge on the usage of cannabinoid substances among cancer patients receiving systemic anti-cancer treatment in the Netherlands. The current study revealed that almost a quarter of the cancer patients have used cannabinoid substances for medical purposes, with a prevalence of active users of 15%. Potential future medical usage by current non-users was reported to be 23%. CBD-oil was the most frequently reported way of consumption. Users consisted mainly of patients undergoing treatment with palliative intent. More than half of the patients reporting use of cannabinoid substances, received immunotherapy treatment. Intention of treatment, as well as type of therapy, turned out to be predictive factors for cannabinoid consumption. Although motivations for usage varied, about half of the users, reported the supposed anti-cancer properties of cannabinoids as their motivation to engage in consumption of these substances.

The high prevalence of self-reported use of cannabinoid substances among cancer patients in the Netherlands, is in line with the findings from several other studies, suggesting that cannabinoid usage among this patient group should not be underestimated. A Canadian study, performed prior to the Canadian Cannabis Act, reported a similar prevalence of active users of 18% (Martell et al. 2018). We expected the cannabinoid usage in our study to be higher than reported in the Canadian study, for several reasons. First of all, the current study is the first study selecting patients receiving systemic therapy, which was a predictor for cannabinoid consumption in the Canadian study (Martell et al. 2018). Furthermore, in Canada consumption of cannabis for medical purposes was an exemption under the law prior to Canadian Cannabis Act in 2018 (Martell et al. 2018), while in the Netherlands both recreational as well as medical use of cannabis are tolerated though not legalized (Gielen and de Vrey 2021). This would justify higher prevalence of users in the current study compared to the Canadian study. Pergam et al. (2017), who performed a similar study in Washington, where cannabis has been fully legalized, revealed a prevalence of active users of 24% (Pergam et al. 2017). Although cultural differences between the USA and the Netherlands could affect the prevalence of consumption, a more likely explanation is the methodology of both studies. In the Pergam study a considerable percentage of users reported exclusive recreational use, whereas our study allowed for specifying consumption based on medical intent. The innovative aspect of the current study, including the selection of patients receiving systemic therapy combined with the potential exclusive focus on consumption of cannabinoid substances with medical intent, contributes to the differences in prevalence found between the studies and allows for a conscientious appraisal of medical cannabinoid consumption among cancer patients in the Netherlands receiving systemic therapy, which turned out to be clinically significant.

The prevalence of cannabinoid users we found in the current study, is considerably higher than the reported prevalence rate of prescribed cannabis in the general population (Stichting Farmaceutische Kengetallen 2021). This difference is mainly due to the fact that most patients rely on a different resource for their cannabinoids, as also reported in the current study. Additionally, the prevalence found in the current study, is higher than the most recently reported yearly prevalence of cannabis in the general population (7.5%) of the Netherlands (van Laar and van Gestel 2019), especially when specified for the age group of the current study (1.8%) (Trimbos Instituut 2017). These findings imply an increased susceptibility to engagement in consumption of cannabinoids among the oncology patients within this age group.

The consumption of cannabinoids by cancer patients is significant, particularly when compared to the general population. The observation that over half of the patients that reported active use of cannabinoids, concurrently received immunotherapy, may be of concern. The immunomodulatory role of cannabinoid substances in cancer is not yet clear and their safety during immunotherapy is not guaranteed. Recent data from a prospective observational study by Bar-Sela et al., reported decreased time to progression and shorter overall survival for cannabis-users receiving immunotherapy treatment (Bar-Sela et al. 2020). Additionally, in an earlier retrospective observational study of Taha et al. (2019), consumption of cannabis was associated with reduced response rates to Nivolumab in patients with advanced cancer (Taha et al. 2019). Underlying mechanisms concern the number and functioning of available lymphocytes, which might be altered through exposure to cannabinoids (Bar-Sela et al. 2020). These findings suggest that adjunctive treatment with cannabinoids should be approached with caution.

A commonly reported symptom for consumption of cannabinoids, was the treatment of pain, which could imply inadequate pain management or pain refractory to commonly used pain medication, including opioids. Although in total only 4 consumers reported an NRS score equal or higher than 5, information about additional pain medication was incomplete. Despite the limited evidence, the Dutch Guideline for Policy and Treatment of Pain in Cancer, advises to consider consumption of cannabinoids in pain refractory to other pain medication (Federatie Medisch Specialisten 2019). The consumption of cannabinoids to manage pain, is in line with previous studies, where also mainly the expected analgesic effect was reported as the motivation for usage (Donovan et al. 2019; Mousa et al. 2019; Pergam et al. 2017). However, whereas in other studies particularly THC was consumed (Martell et al. 2018; Mousa et al. 2019; Pergam et al. 2017), the current study reported CBD-oil as the most common way of consumption. However, THC is proposed to be the analgesic component in cannabinoid substances (Good et al. 2019; Hardy et al. 2020; MacDonald and Farrah 2019) (Whiting et al. 2015), rather than the CBD-component. Strikingly, of the patients who used the cannabinoids to treat pain, only 50% used a THC compound.

Another great concern is the observation of the assumed anti-cancer effect of cannabinoids as driving motivation for use. With 46%, the percentage of patients in our study reporting this potential therapeutic effect as a reason for consumption, is higher than found in some previous studies, where values around 25–30% have been reported (Mousa et al. 2019; Pergam et al. 2017). Not only current or previous users adhere to this therapeutic believe of cannabinoids, but also a remarkable 20% of non-users reported considering using cannabinoids because of its proposed anti-cancerous effect. To date no results of large clinical trials have been published supporting the use of cannabinoids for anti-cancer purposes. Rather, potential evidence regarding effectivity has been limited to in vitro and in vivo studies (Abrams and Guzman 2015; Bouquié et al. 2018; Daris et al. 2019; Turgeman and Bar-Sela 2017). The role of social media seems to be a compelling factor in creating this believe, with news stories claiming cannabis as an alternative treatment to cure cancer being widely spread (Shi et al. 2019).

The persisting profound believe in the assumed anti-cancerous effect in absence of clinical evidence, could be related to presumed unawareness by the clinicians regarding consumption of cannabinoids by their patients. This is in line with the low rates of reported usage in the patient files found in our study, underlining the lack of active involvement of the clinician towards this topic. This suggests that even in a country where cannabis consumption is relatively tolerated, cannabinoid consumption as alternative or adjunctive treatment is not yet a topic which is easily addressed by either the clinician or the patient, which is in agreement with previous research (Braun et al. 2020; Kleckner et al. 2019; Pergam et al. 2017). Absence of clinical guidance, however, leaves the patients relying on non-medical sources of information, with presumed lower degrees of clinical evidence (Zolotov et al. 2021), thereby potentially posing patients at medical risks. This does not only concern the anti-cancer believe, but also the potential risks related to concurrent immunotherapy. Furthermore, self-medicating might lead to consumption of products for purposes which are not actually served by the substance. Additionally, it increases the risk of overdosing products, leads to a higher risk of dependency (Fitzcharles and Eisenberg 2017; Hazekamp and Pappas 2014) and increases the risk of using polluted products. The startling amount of users among cancer patients in absence of clinical guidance, combined with patients’ presumed misperception regarding its purposes and utility, as shown in the current study, stresses the need for a change. It necessitates a diligent role of the clinician and addresses the urgency of adequate patient education on both the potential therapeutic benefits, as well as the eventual risks, of cannabinoid consumption in cancer (Donovan et al. 2019). This is especially important in light of the high number of users receiving immunotherapy, as revealed in the current study.

Considering the observational cross-sectional design, this study has its limitations. Even though different types of tumors are represented in the study, the study might not completely reflect the diversity of tumors as presented in the general population, as a result of using a convenience sample, thereby increasing the risk of selection bias. Furthermore, even though the response rate might have been increased through the support of the researcher while filling in the questionnaire, it could have negatively affected the report of usage due to social desirability, thereby underreporting the actual prevalence of cannabinoid usage. Finally, statistical analysis was applied on a small sample size. Even though the study should be interpreted in light of these intrinsic limitations of the study, this study greatly emphasizes the uncertainties and difficulties regarding the current issue of cannabinoid usage among cancer patients.

### Recommendations for further research

To encourage clinical guidance, this study comes with some recommendations for further research. First of all, more research is needed on patients’ perception concerning usage of cannabinoid substances. Up until now, relatively little is known about the clinical processes of decision making by patients, including how they access information (Braun et al. 2020). Additional research in the form of semi-structured interviews, would enable better understanding of patients’ perceptions of cannabis’ therapeutic effects, as well as its adverse effects, and creates awareness regarding their considerations for usage (Donovan et al. 2020; Zarrabi et al. 2019). This would aid clinicians in finding an effective dialog with patients with respect to this topic. Additionally, research on the safety and efficacy of medical cannabis is encouraged, to provide the doctors with adequate information to enable them to competently guide their patients, since many health care professionals reported to feel unprepared for this topic (Arboleda et al. 2020; Braun et al. 2020; Zolotov et al. 2021). Semi-structured interviews with medical specialist would be helpful in assessing their current beliefs. This is especially important in light of the great discordance between patient-perceived effects of cannabinoids and those effects retrieved from clinical trials, as also seen in the current study (Aviram et al. 2020; Good et al. 2019; Hardy et al. 2020).

## Conclusion

This study underlines the high rates cannabinoid use among cancer patients in the Netherlands and the existing challenges regarding motivation for usage, consumption characteristics and potential interaction with concurrent treatment. It also shows that simultaneously, the awareness among medical professionals regarding cannabinoid consumption by their patients is disturbingly low. This study highlights the importance of a pro-active role of the clinician, assessing usage of cannabinoid substances and adequately educating the patients on potential therapeutic benefits and risks, thereby preventing reliance on non-medical sources. Since data on efficacy and safety of cannabinoids is currently ambiguous, more research is required to enable competent patient education. Furthermore, additional research on attitudes of patients and their decision-making process through semi-structured interviews is recommended, to improve adequate clinical guidance.

## Supplementary Information

Below is the link to the electronic supplementary material.

Supplementary file1 (DOCX 22 KB)

Supplementary file2 (PDF 2010 KB)

## Data Availability

Datasets are available from the corresponding author on reasonable request.
