# Supplementary material for: Cannabinoid consumption among cancer patients receiving systemic anti-cancer treatment in the Netherlands
Source: J Cancer Res Clin Oncol. 2022 Jul 2;149(5):1863–72. doi: 10.1007/s00432-022-04085-z (PMC10097765; doi:10.1007/s00432-022-04085-z)
Supplement: Supplementary file 1 — Supplementary file1 (DOCX 22 KB) [file 432_2022_4085_MOESM1_ESM.docx]

**Cannabinoid consumption among cancer patients receiving systemic anti-cancer treatment in the Netherlands**

Y. Oelen^1^, S. Revenberg^1^, J. de Vos-Geelen^1^, R. van Geel^2,3^, J. Schoenmaekers^3,4^, M. van den Beuken-Everdingen^4^, L.B.J. Valkenburg-van Iersel^1^

Affiliations of authors:

^1^ Division of Medical Oncology, Department of Internal Medicine, GROW-School for Oncology and Developmental Biology, Maastricht University Medical Center, Maastricht, the Netherlands.

^2^ Department of Clinical Pharmacy and Toxicology, Maastricht University Medical Center+, Maastricht, the Netherlands

^3^ CARIM School for Cardiovascular Disease, Maastricht University, Maastricht, the Netherlands

^4^ Centre of Expertise for Palliative Care, Maastricht University Medical Centre (MUMC+), Maastricht, the Netherlands

**Journal:** Journal of Cancer Research and Clinical Oncology

**e-mail address:** yrina.oelen@mumc.nl

**APPENDIX 1 – Reported symptoms for consumption among separated for previous and current users with medical intent**

**Fig. 3** Reported reasons for use of cannabinoids with medical intention, among previous users. Represented in absolute values.

**Fig. 4** Reported reasons for use of cannabinoids with medical intention, among current users. Represented in absolute values
