# Supplementary material for: Cannabinoid consumption among cancer patients receiving systemic anti-cancer treatment in the Netherlands
Source: J Cancer Res Clin Oncol. 2022 Jul 2;149(5):1863–72. doi: 10.1007/s00432-022-04085-z (PMC10097765; doi:10.1007/s00432-022-04085-z)
Supplement: Supplementary file 2 — Supplementary file2 (PDF 2010 KB) [file 432_2022_4085_MOESM2_ESM.pdf]

Interview / enquête Cannabis gebruik bij patiënten met kanker (versie nov 2020)

Patiëntcode:

ALGEMEEN

1. Geslacht
   ☐ Man
   ☐ Vrouw

2. Leeftijd:

3. Hoogst genoten opleidingsniveau
   ☐ Basisschool
   ☐ Middelbare school
   ☐ MBO
   ☐ HBO
   ☐ Academisch
   ☐ Post-academisch

4. Etnische achtergrond:

5. Diagnose maligniteit:
   ☐ Mamma
   ☐ Gastro-intestinaal (GE)
   ☐ Urogenitaal
   ☐ Pulmonaal
   ☐ Sarcoom
   ☐ Melanoom
   ☐ Gynaecologisch
   ☐ Hoofd/hals
   ☐ Anders, namelijk:

6. Behandelintentie:
   ☐ Curatief
   ☐ Palliatief

7. Behandeling:
   a. ☐ Chemotherapie ☐ Immunotherapie ☐ Targeted therapy, namelijk:
   b. ☐ Adjuvant ☐ Neo-adjuvant ☐ 1^{e} lijn ☐ 2^{e} lijn ☐ ≥ 3^{e} lijn

8. Comorbiditeit:
   ☐ Diabetes Mellitus
   ☐ Pulmonaal
   ☐ Cardiovasculair
   ☐ Psychiatrisch
   ☐ Anders, namelijk:

9. Huidige medicatie:

    - ❖
    - ❖
    - ❖
    - ❖
    - ❖
    - ❖
    - ❖
    - ❖
    - ❖
    - ❖

10. Rookgeschiedenis
    ☐ Huidige roker, packyears:
    ☐ Vroeger gerookt, packyears:
    ☐ Nooit gerookt

CANNABIS GEBRUIK

11. Gebruikt u nu, of heeft u in het verleden een cannabis product gebruikt?
    ☐Nee, nooit gebruikt ga naar vraag 12.
    ☐Ja in verleden, recreatief ga naar vraag 17.
    ☐Ja in verleden, medicinaal ga naar vraag 22.
    ☐Ja momenteel, recreatief ga naar vraag 26.
    ☐Ja momenteel, medicinaal ga naar vraag 34.

12. Overweegt u een cannabis product te gaan gebruiken?
    ☐Ja — ga naar vraag 12.
    ☐Nee — deze enquête / interview is niet op u van toepassing

13. Hoe heeft u van cannabis producten gehoord?
    ☐Via vrienden / kennissen / familie
    ☐Via internet
    ☐Via lotgenoten
    ☐Via mijn arts / verpleegkundige
    ☐Anders, namelijk:

14. Welke soort cannabis product overweegt u te gaan gebruiken?
    ☐CBD olie ……%
    ☐CBD/THC olie …….. / …….. %
    ☐THC olie ……%
    ☐Cannabis thee CBD.......% / ....THC........%
    ☐Cannabis roken / vernevelen CBD.......% / ....THC........%
    ☐Weet ik niet
    ☐Anders, namelijk:

15. Waar wil u het cannabis product aanschaffen?
    ☐Bij drogisterij of apotheek zonder recept
    ☐Via coffee shop
    ☐Via vrienden/familie
    ☐Eigen kweek
    ☐Op recept via arts
    ☐Via internet
    ☐Weet ik nog niet
    ☐Anders, namelijk:

16. Voor welke klachten / ziekten overweegt u een cannabis product te gaan gebruiken? *(meerdere antwoorden mogelijk)*
    ☐ADHD
    ☐Pijn
    ☐Zenuwpijn
    ☐Spasmen
    ☐Angst
    ☐Depressie
    ☐Stress
    ☐Eetlust
    ☐Misselijkheid
    ☐Overgeven

☐Slapeloosheid/slaapstoornis
☐Tics
☐Verhoogde oogdruk (glaucoom)
☐HIV/aids
☐Kanker (genezend)
☐Multiple sclerose (genezend)
☐Anders, namelijk:

Einde enquête

17. Overweegt u nu of in de nabije toekomst een cannabis product te gaan gebruiken voor medicinale doeleinden?

☐Ja	ga naar vraag 17.
☐Nee	deze enquête / interview is niet op u van toepassing

18. Voor welke klachten / ziekten overweegt u een cannabis product te gaan gebruiken? *(meerdere antwoorden mogelijk)*

☐ADHD
☐Pijn
☐Zenuwpijn
☐Spasmen
☐Angst
☐Depressie
☐Stress
☐Eetlust
☐Misselijkheid
☐Overgeven
☐Slapeloosheid/slaapstoornis
☐Tics
☐Verhoogde oogdruk (glaucoom)
☐HIV/aids
☐Kanker (genezend)
☐Multiple sclerose (genezend)
☐Anders, namelijk:

19. Hoe heeft u van cannabis producten voor medicinale toepassingen gehoord?

☐Via vrienden / kennissen / familie
☐Via internet
☐Via lotgenoten
☐Via mijn arts / verpleegkundige
☐Anders, namelijk:

20. Welk soort cannabis product overweegt u te gaan gebruiken?

☐CBD olie	……%
☐CBD/THC olie	…….. / …….. %
☐THC olie	……%
☐Cannabis thee	CBD…….% / ……..THC……..%
☐Cannabis roken / vernevelen	CBD…….% / ……..THC……..%
☐Weet ik niet	
☐Anders, namelijk:	

21. Waar wil u het cannabis product aanschaffen?
    ☐Bij drogisterij of apotheek zonder recept
    ☐Via coffee shop
    ☐Via vrienden/familie
    ☐Eigen kweek
    ☐Op recept via arts
    ☐Via internet
    ☐Weet ik nog niet
    ☐Anders, namelijk:

Einde Enquête

22. Voor welke indicatie gebruikte u een cannabis product? (*Meerdere antwoorden mogelijk)*
    Geef het door u ervaren effect een score 1-4 (1= geen effect; 4= maximaal effect)
    ☐ADHD
    ☐Pijn
    ☐Zenuwpijn
    ☐Spasmen
    ☐Angst
    ☐Depressie
    ☐Stress
    ☐Eetlust
    ☐Misselijkheid
    ☐Overgeven
    ☐Slapeloosheid/slaapstoornis
    ☐Tics
    ☐Verhoogde oogdruk (glaucoom)
    ☐HIV/aids
    ☐Kanker (genezend)
    ☐Multiple sclerose (genezend)
    ☐Anders, namelijk:

23. Welk soort cannabis product gebruikte u?
    ☐CBD olie ……%
    ☐CBD/THC olie …….. / …….. %
    ☐THC olie ……%
    ☐Cannabis thee CBD…….% / ……..THC……..%
    ☐Cannabis roken / vernevelen CBD…….% / ……..THC……..%
    ☐Weet ik niet
    ☐Anders, namelijk:

24. Hoe lang heeft u een cannabis product gebruikt?
    ………………maanden/jaren (doorhalen wat niet van toepassing is)

25. Waarom bent u gestopt met het gebruik van het cannabis product? *(meerdere antwoorden mogelijk)*
    ☐Geen werking
    ☐Te veel bijwerkingen
    ☐Te duur
    ☐Mijn arts raadde het af
    ☐Anders, namelijk:

Einde enquête

26. Welk soort cannabis product gebruikt u?
    ☐CBD olie ……%
    ☐CBD/THC olie …….. / …….. %
    ☐THC olie ……%
    ☐Cannabis thee CBD.......% / ....THC........%
    ☐Cannabis roken / vernevelen CBD.......% / ....THC........%
    ☐Weet ik niet
    ☐Anders, namelijk:

27. Hoe lang gebruikt u reeds een cannabis product?
    ……………….maanden/jaren (doorhalen wat niet van toepassing is)

28. Hoe vaak gebruikt u het cannabis product?
    ☐< 1x / week
    ☐1 – 5x/ week
    ☐Dagelijks
    ☐Meerdere malen per dag :…………… keer
    ☐Zeg ik liever niet

29. Hoeveel van het cannabis product gebruikt u per keer?
    ☐Aantal druppels:
    ☐Aantal joints:
    ☐Anders, namelijk:
    ☐Zeg ik liever niet

30. Waar haalt u uw cannabis product?
    ☐Bij drogisterij of apotheek zonder recept
    ☐Via coffee shop
    ☐Via vrienden/familie
    ☐Eigen kweek
    ☐Op recept via arts
    ☐Via internet
    ☐Weet ik nog niet
    ☐Anders, namelijk:

31. Heeft het gebruik van het cannabis product ook effecten op klachten als gevolg van de kanker?
Geef het door u ervaren effect een score 1-4 (1= geen effect; 4= maximaal effect)

**Klacht**		**Niet van toepassing / weet niet**
Pijn		
Zenuwpijn		
Misselijkheid		
Overgeven		
Jeuk		
Eetlust		
Vermoeidheid		
Gewicht		
Depressie		
Angst		
Stress		
Slapeloosheid		

32. Indien het cannabis product helpt voor een van eerder genoemde klachten: hoe snel werkt het?
............minuten niet van toepassing

33. Indien het cannabis product helpt voor een van eerder genoemde klachten: hoe lang werkt het?
............minuten niet van toepassing

Einde enquête

34. Welk soort cannabis product gebruikt u?
    - ☐CBD olie ……%
    - ☐CBD/THC olie …….. / …….. %
    - ☐THC olie ……%
    - ☐Cannabis thee CBD.......% / ....THC........%
    - ☐Cannabis roken / vernevelen CBD.......% / ....THC........%
    - ☐Weet ik niet
    - ☐Anders, namelijk:

35. Hoe lang gebruikt u reeds een cannabis product?
    ……………….maanden/jaren (doorhalen wat niet van toepassing is)

36. Hoe vaak gebruikt u het cannabis product?
    - ☐< 1x / week
    - ☐1 – 5x/ week
    - ☐Dagelijks
    - ☐Meerdere malen per dag :…………… keer
    - ☐Zeg ik liever niet

37. Hoeveel van het cannabis product gebruikt u per keer?
    - ☐Aantal druppels:
    - ☐Aantal joints:
    - ☐Anders, namelijk:
    - ☐Zeg ik liever niet

38. Waar haalt u uw cannabis product?
    - ☐Bij drogisterij of apotheek zonder recept
    - ☐Via coffee shop
    - ☐Via vrienden/familie
    - ☐Eigen kweek
    - ☐Op recept via arts
    - ☐Via internet
    - ☐Weet ik nog niet
    - ☐Anders, namelijk:

39. Waar gebruikt u het cannabis product voor?
    a. Als anti-kanker medicijn ja / nee
    b. Tegen lichamelijke klachten ja / nee
    c. Tegen psychische klachten ja / nee

40. Voor welke klachten gebruikt u het cannabis product, en helpt het?
    Geef het door u ervaren effect een score 1-4 (1= geen effect; 4= maximaal effect)

**Klacht**		**Niet van toepassing / weet niet**
Pijn		
Zenuwpijn		
Misselijkheid		
Overgeven		
Jeuk		
Eetlust		
Vermoeidheid		
Gewicht		
Depressie		
Angst		
Stress		
Slapeloosheid		

41. Indien het cannabis product helpt voor een van eerder genoemde klachten: hoe snel werkt het?
    ....minuten niet van toepassing

42. Indien het cannabis product helpt voor een van eerder genoemde klachten: hoe lang werkt het?
    ....minuten niet van toepassing

Einde enquête

Cannabinoid consumption among cancer patients receiving systemic anti-cancer treatment in the Netherlands

Y. Oelen1, S. Revenberg1, J. de Vos-Geelen1, R. van Geel2,3, J. Schoenmaekers3,4, M. van den Beuken-Everdingen4, L.B.J. Valkenburg-van Iersel1

Affiliations of authors:
1 Division of Medical Oncology, Department of Internal Medicine, GROW-School for Oncology and Developmental Biology, Maastricht University Medical Center, Maastricht, the Netherlands.

2 Department of Clinical Pharmacy and Toxicology, Maastricht University Medical Center+, Maastricht, the Netherlands

3 CARIM School for Cardiovascular Disease, Maastricht University, Maastricht, the Netherlands

4 Centre of Expertise for Palliative Care, Maastricht University Medical Centre (MUMC+), Maastricht, the Netherlands

Journal: Journal of Cancer Research and Clinical Oncology
e-mail address: yrina.oelen@mumc.nl
